# Circulating CD8^+^CD122^+^ T cells as a prognostic indicator of pancreatic cancer

**DOI:** 10.1186/s12885-022-10207-0

**Published:** 2022-11-04

**Authors:** Katsuhito Teramatsu, Takamasa Oono, Koki Oyama, Nao Fujimori, Masatoshi Murakami, Sho Yasumori, Akihisa Ohno, Kazuhide Matsumoto, Ayumu Takeno, Kohei Nakata, Masafumi Nakamura, Yoshihiro Ogawa

**Affiliations:** 1 Department of Medicine and Bioregulatory Science, Graduate School of Medical Sciences, Kyushu University, 3-1-1, Maidashi, Higashi-ku, Fukuoka, 812-8582 Japan; 2 Department of Hepatology & Pancreatology, Kyushu University Hospital, 3-1-1, Maidashi, Higashi-ku, Fukuoka, 812-8582 Japan; 3 Department of Surgery and Oncology, Graduate School of Medical Sciences, Kyushu University, 3-1-1, Maidashi, Higashi-ku, Fukuoka, 812-8582 Japan

**Keywords:** Metastatic pancreatic cancer, Resectable pancreatic cancer, Benign pancreatic cysts, CD4^+^ T cells, CD8^+^CD122^+^ T cells

## Abstract

**Purpose:**

The distribution of tissue infiltrating lymphocytes has been shown to affect the prognosis of patients with pancreatic cancer in some previous studies. However, the role of peripheral lymphocytes in pancreatic cancer remains debated. The purpose of this study was to analyze the peripheral subtypes of T lymphocytes, and establish their association with the prognosis of patients with pancreatic cancer.

**Methods:**

Blood and tissue samples were collected from patients with metastatic pancreatic cancer (*n* = 54), resectable pancreatic cancer (*n* = 12), and benign pancreatic cysts (*n* = 52) between April 2019 and January 2022 and analyzed.

**Results:**

Patients with metastatic pancreatic cancer had a larger proportion of both tumor-suppressive and tumor-promoting cells than those with benign pancreatic cysts. In addition, the proportion of peripheral CD4^+^ T cells positively correlated with the survival of patients with metastatic pancreatic cancer, and the proportion of peripheral CD8^+^CD122^+^ T cells was associated with early mortality (< 90 days). After chemotherapy, CD8^+^CD122^+^ T cells decreased in patients who had a partial response or stable disease. Moreover, by analyzing resected specimens, we first proved that the existence of CD8^+^CD122^+^ T cells in a tumor microenvironment (TME) depends on their proportion in peripheral blood.

**Conclusion:**

Circulating CD8^+^CD122^+^ T cells can be a prognostic indicator in patients with pancreatic cancer.

**Supplementary Information:**

The online version contains supplementary material available at 10.1186/s12885-022-10207-0.

## Introduction

Pancreatic cancer has the highest mortality rate among gastrointestinal cancers, and it is the fourth leading cause of cancer-related deaths worldwide [1]. The high mortality rate is due to the difficulty of early diagnosis and poor response to chemotherapy. Approximately 80% of patients are diagnosed with unresectable disease at the time of onset [2]. Although modern intense chemotherapy regimens such as FOLFIRINOX and gemcitabine plus nab-paclitaxel (GnP) have improved the survival rate of patients with metastatic pancreatic cancer; survival time remains limited, with a median of 8.5–11.1 months [3, 4].

Recently, immunotherapy has been regarded as an effective option for various cancer types such as malignant melanoma, non-small cell lung cancer, and renal cell carcinoma. However, in pancreatic cancer, the antitumor effects of immunotherapy have been unsatisfactory [5, 6]. Low cancer immunogenicity and a unique tumor microenvironment (TME), characterized by an enriched stroma and many immunosuppressive cells, are thought to result in poor treatment responses. In the TME, CD8^+^ T cells, natural killer (NK) cells, and M1-polarized macrophages (M1Mφ) act as tumor-suppressive cells, preventing cancer cell growth and eliminating cancer cells. In contrast, regulatory T cells (Tregs), M2-polarized macrophages (M2Mφ), and myeloid-derived suppressor cells (MDSC) form an immunosuppressive network that inhibits other effector T cells and NK cells, thereby promoting immune evasion of the tumor [5, 7, 8].

The distribution of tissue infiltrating lymphocytes (TILs) has been previously shown to affect the prognosis of patients with pancreatic cancer [9]. In previous studies using resected specimens of pancreatic cancer, the number of CD8^+^ T cells in the TME positively correlated with patient survival, whereas the numbers of Tregs and M2Mφs were associated with poor prognoses [9–12]. Indeed, in patients with both resectable and unresectable cancers, peripheral blood CD8^+^ T cells positively correlated, while CD4^+^ Tregs negatively correlated with survival rate [13–15]. Additionally, the distribution of peripheral blood lymphocytes (PBLs) and TILs was confirmed to be a predictive factor for survival in patients with pancreatic cancer [16]. Many unknowns remain regarding the role of PBLs in pancreatic cancer.

Similar to classical CD4^+^ Tregs, CD8^+^ T cells include both regulatory and effector subtypes. The regulatory subtype of CD8^+^ T cells has been regarded as an immunosuppressive subtype of T lymphocytes, which play a vital role in self-tolerance [17]. Early studies reported that regulatory CD8^+^ T cells prevented autoimmune diseases [18–20] and controlled the rejection of allografts and graft-versus-host disease (GVHD) during organ transplantation [21–27]. In a few reports, regulatory CD8^+^ T cells were found to mediate antitumor immunity in the presence of cancer. Li et al. (2011) revealed that CD8^+^FOXP3^+^ T cells were increased in the peripheral blood of patients with nasopharyngeal cancer compared with healthy donors, and these cells infiltrated the TME [28]. Kiniwa et al. (2007) demonstrated that CD8^+^FOXP3^+^ T cells derived from TILs of patients with prostate cancer suppressed naïve T cell proliferation in vitro [29]. Nevertheless, the mechanisms through which regulatory CD8^+^ T cells influence tumor progression or prognosis remain unknown.

In this study, we investigated the subtypes of PBLs, including effector and regulatory subtypes of CD8^+^ T cells, as prognostic indicators in patients with metastatic pancreatic cancer (MPC). In addition, we collected tissue and blood samples from patients diagnosed with resectable pancreatic cancer (RPC) to compare the distribution of PBLs with that of TILs. Moreover, to compare the distribution of PBLs of patients diagnosed with MPC with that of patients with a benign pancreatic disease, we therefore collected blood samples from patients with benign pancreatic cysts(BPC).

## Materials and methods

### Patients and sample collection

Blood samples were collected in heparinized tubes from patients with MPC (*n* = 54), RPC (*n* = 12), and BPC (*n* = 52) between April 2019 and January 2022. All patients with MPC and RPC were histologically verified; patients with MPC were treatment-naïve and had no other cancers. Blood samples were collected from patients with MPC before the introduction and 2 months after. Blood samples from patients with RPC were collected immediately before surgery, and tissue samples were obtained from the resected specimens. Patients with MPC received S-IROX, modified FOLFIRINOX (mFFX), or GnP. S-IROX was administered in a clinical trial. After receiving S-IROX or mFFX, patients were treated every 2 weeks: S^− 1^ 40 mg/m^2^ was administered orally twice daily on days 1 to 7 in S-IROX, and 5-fluorouracil 2400 mg/m^2^ was intravenously administered for 46 h without bolus infusion in mFFX, in addition to intravenous oxaliplatin (85 mg/m^2^) and irinotecan (150 mg/m^2^) on day 1. Patients in GnP therapy received gemcitabine 1000 mg/m^2^ plus nab-paclitaxel 125 mg/m^2^ intravenously on days 1, 8, and 15 of each 28-day cycle. Computed tomography (CT) or magnetic resonance imaging (MRI) was used to evaluate the response to chemotherapy every 2–3 months according to the guidelines of the Response Evaluation Criteria in Solid Tumors (RECIST) 1.0. Patients with BPC had serous cyst neoplasm (SCN), simple cyst, or intraductal papillary mucinous neoplasm (IPMN), but no malignant findings in the pancreas, as evaluated by image inspection such as CT, MRI, and endoscopic ultrasonography. The study protocol was approved by the Ethics Committee of Kyushu University, and informed consent was obtained in writing from all patients (approval number, 2020–620).

### Processing tissue samples

Tissue samples were washed with DMEM (high glucose) (FUJIFILM Wako Pure Chemical Corporation, Japan) and minced into pieces < 0.75 μm in length. BD HorizonTM Dri Tumor and Tissue Dissociation Reagent (TTDR) (BD Biosciences, Tokyo, Japan) was used to extract TILs from tissues. According to the protocol presented, minced tissues were suspended in 1x TTDR and incubated at 37 °C with frequent agitation for 75 min. Three volumes of phosphate-buffered saline (PBS) were added to the TTDR, including tissues, and filtered through a 70-μm filter (Falcon Catalog number 352350). The filtrate was centrifuged at 300×g for 8 min. The pellets, including TILs, were suspended in cell-staining buffer (BioLegend, Tokyo, Japan) and used for flow cytometry.

### Flow cytometry

Peripheral blood mononuclear cells (PBMCs) were purified from each venous blood sample by Lymphoprep™ (Serunwerk Bernburg, Oslo, Norway) and washed twice with PBS. PBMCs and TILs, collected as described above, were suspended in cell-staining buffer and FcR blocking reagent (Miltenyi Biotec, Bergisch Gladbach, Germany) was added to block non-specific binding for 10 min. PBMCs and TILs were stained for cell surface molecules, while PBMCs were intracellularly stained for Foxp3 and cytotoxic T-lymphocyte-associated protein 4 (CTLA4). The used antibodies (Abs) are follows: Brilliant Violet 421™ anti-CD122 (clone TU27, BioLegend, Tokyo, Japan), Brilliant Violet 605™ anti-CD45R (clone RA3-6B2, BioLegend, Tokyo, Japan), Brilliant Violet 785™ anti-CD4 (clone RPA-T4, BioLegend, Tokyo, Japan), Brilliant Violet 785™ anti-CD8 (clone SK1), fluorescein isothiocyanate (FITC) anti-CD3 (clone OKT3, BioLegend, Tokyo, Japan), FITC anti-CD4(clone RPA-T4, BioLegend, Tokyo, Japan), Pacific Blue™ anti-CD3 (clone OKT3, BioLegend, Tokyo, Japan), phycoerythrin (PE) anti-FOXP3 (clone 150D, BioLegend, Tokyo, Japan), PE anti-CD107a (clone H4A3, BioLegend, Tokyo, Japan), phcoerythrin-cyanine™ 7 (PE-Cy™7) anti-CD28 (clone CD28.2, BioLegend, Tokyo, Japan), Allophycocyanin (APC) anti-CD25 (clone BC96, BioLegend, Tokyo, Japan), APC anti-CD19 (clone HIB19, BioLegend, Tokyo, Japan), Alexa Fluor647™ anti-CTLA4 (clone L3D10, BioLegend, Tokyo, Japan), Brilliant Violet 421™ mouse IgG1κ (clone MOPC-21, BioLegend, Tokyo, Japan), Brilliant Violet 605™ mouse IgG1κ (clone MOPC-21, BioLegend, Tokyo, Japan), Brilliant Violet 785™ mouse IgG1κ (clone MOPC-21, BioLegend, Tokyo, Japan), FITC mouse IgG1κ (clone MOPC-21, BioLegend, Tokyo, Japan), FITC mouse IgG2aκ (clone MOPC-173, BioLegend, Tokyo, Japan), Pacific Blue™ mouse IgG2aκ (clone MOPC-173, BioLegend, Tokyo, Japan), PE mouse IgG1κ(clone MOPC-21, BioLegend, Tokyo, Japan), PE-Cy™ 7 mouse IgG1κ (clone MOPC-21, BioLegend, Tokyo, Japan), APC mouse IgG1κ (clone MOPC-21, BioLegend, Tokyo, Japan), Alexa Fluor^R^ 647 mouse IgG1κ (clone MOPC-21, BioLegend, Tokyo, Japan). We used Zombie NIR™ dye (BioLegend, Tokyo, Japan) to determine cell viability. PBMCs were stained with Ab and Zombie dye on the surface at room temperature in the dark for 20 min. Next, using the Fixation/Permeabilization Buffer Set (eBioscience, Tokyo, Japan), we stained intracellular FOXP3 or CTLA4 using the manufacturer’s instructions. Briefly, each sample was incubated with a fixation/permeabilization buffer at room temperature in the dark for 30 min. Each sample was washed twice with permeabilization buffer, anti-FOXP3 Ab or anti-CTLA4 Ab was added, and the samples were incubated at room temperature in the dark for 30 minutes. Each sample was washed twice with permeabilization buffer, diluted in cell-staining buffer, and analyzed by flow cytometry. All the prepared samples were analyzed using a CytoFLEX flow cytometer (Beckman Coulter, Brea, California, USA). The gating strategy is illustrated in Online Resources 1–3.

### Statistical analysis

Statistical analysis was conducted using the JMP v. 16 software (SAS and JMP, Institute Inc., Cary, NY, USA). We analyzed the differences in each PBL subtype between patients with MPC and BPC using Student’s t-test. To evaluate the correlation between two continuous variables, we performed correlation analysis using Spearman’s rank correlation coefficient. The Kaplan–Meier method was employed with the log-rank test to compare survival rates between the two groups. The chi-square test was used to compare categorical groups. Differences between two values of the same groups were analyzed using paired t-test with Wilcoxon signed-rank test. Multivariate analysis was performed using a multiple logistic regression model or a Cox multivariate proportional hazard regression model. Statistical analysis was performed by statistical experts from the Academic Research Organization at Kyushu University.

## Results

### Clinical characteristics of patients with MPC and BPC

Patients with MPC had a mean age of 64.9 (range 42–79) years (26 males and 28 females), and those with BPC a mean age of 65.2 (range 44–76) years (25 males and 27 females). Patients with BPC included 1.9% (1/52) with SCN, 23.1% (12/52) with simple cysts, and 75.0% (39/52) with IPMN. Diabetes mellitus was significantly more common in patients with MPC compared to BPC (42.6%(23/54) vs. 23.1%(12/52), *p* = 0.038), and the serum albumin levels of patients with MPC were significantly lower than those of patients with BPC (*p* < 0.0001). Regarding chemotherapy regimens, 3.7% (2/54), 20.4% (11/54), and 75.9% (41/54) of patients with MPC received S-IROX, modified FOLFIRINOX (mFFX), and GnP, respectively. Patient characteristics are summarized in Table 1.

**Table 1:** Clinical characteristics of patients with metastatic pancreatic cancer and benign pancreatic cysts

Characteristic	Metastatic pancreatic cancer	Benign pancreatic cyst	***P***-value
Total number	54	52	
Sex (%)			
Male	26 (48)	25 (48)	0.99
Female	28 (52)	27 (52)	
Age (years)			
Mean ± SD	64.9 ± 8.9	65.2 ± 7.6	0.85
Range	42–79	44–76	
Diabetes mellitus (%)			
Yes	23 (43%)	12 (23%)	**0.038**
Serum albumin (g/dL)			
Median	3.7	4.3	**<0.0001**
Range	2.2–4.5	3.6–5.0	
ECOG performance status (%)			
0/1/2	31(57)/21(39)/2(4)		
1^st^ line chemotherapy regimen (%)			
S-IROX/mFFX/GnP	2(3.7)/11(20.4)/41(75.9)		
T factor (%)			
1/2/3/4	0/0/31(57)/23(43)		
Metastasis location (%)			
Liver/Lung/Peritoneum/Bone	32(59)/15(28)/17(31)/4(7)		
CA19-9 level (U/mL)			
Median	585.1		
Range	1.5–606320		

*ECOG* Eastern Cooperative Oncology Group, *GnP* gemcitabine plus nab-paclitaxel, *SD* standard deviation, *mFFX* modified FOLFIRINOX

*P values <* 0.05 are shown in **bold**

### Proportion of tumor-suppressive and tumor-promoting cells in MPC was higher than that in BPC

**Fig. 1:**
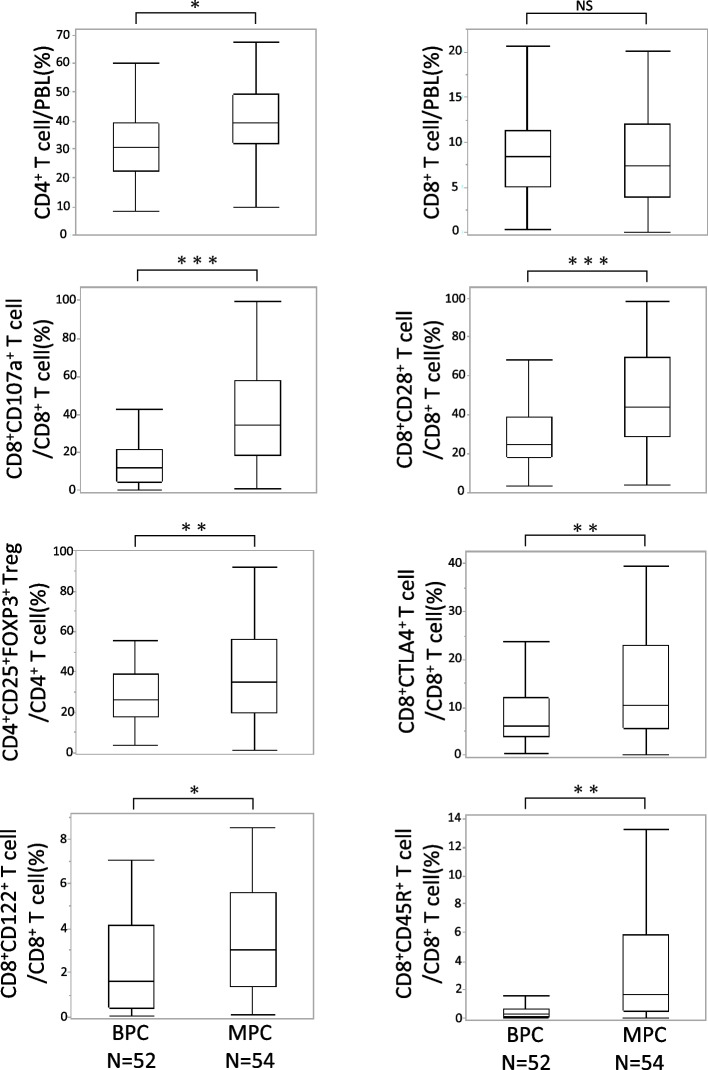
Comparison of the distribution of each subtype of PBLs between BPC and MPC patients using Student’s t-test. *P < 0.05, **P < 0.01, ***P < 0.001, NS: not significant

In the TME, CD8^+^ T cells, NK cells, and M1-polarized macrophages (M1Mφ) are referred to as tumor-suppressive cells, which prevent cancer cell growth and lead to their elimination. In contrast, Tregs, M2Mφ, and MDSC are tumor-promoting cells, leading to cancer cell proliferation and progression [16]. The subtypes of PBLs were determined using flow cytometry with CD4^+^ T cells (CD3^+^CD4^+^), CD8^+^ T cells (CD3^+^CD8^+^), CD4^+^ Tregs (CD4^+^CD25^+^FOXP3^+^), cytotoxic CD8^+^ T cells (CD8^+^CD107a^+^), activated CD8^+^ T cells (CD8^+^CD28^+^), exhausted CD8^+^ T cells (CD8^+^CTLA4^+^), and regulatory CD8^+^ T cells (CD8^+^CD122^+^, CD8^+^CD45R^+^). The PBLs of patients with MPC had a significantly higher proportion of tumor-suppressive cytotoxic CD8^+^CD107a^+^ T cells and activated CD8^+^CD28^+^ T cells (Fig. 1) more than in patients with BPC. Furthermore, PBLs from patients with MPC showed a greater proportion of CD4^+^CD25^+^FOXP3^+^ Tregs, exhausted CD8^+^CTLA4^+^ T cells, CD8^+^CD122^+^ T cells, and CD8^+^CD45R^+^ T cells, which can be regarded as tumor-promoting cells, than in patients with BPC. Thus, patients with MPC had a higher proportion of both tumor-suppressive and tumor-promoting PBLs than patients with BPC.

### CD4^+^ T cell ratio before chemotherapy was related to the prognosis of MPC

**Fig. 2:**
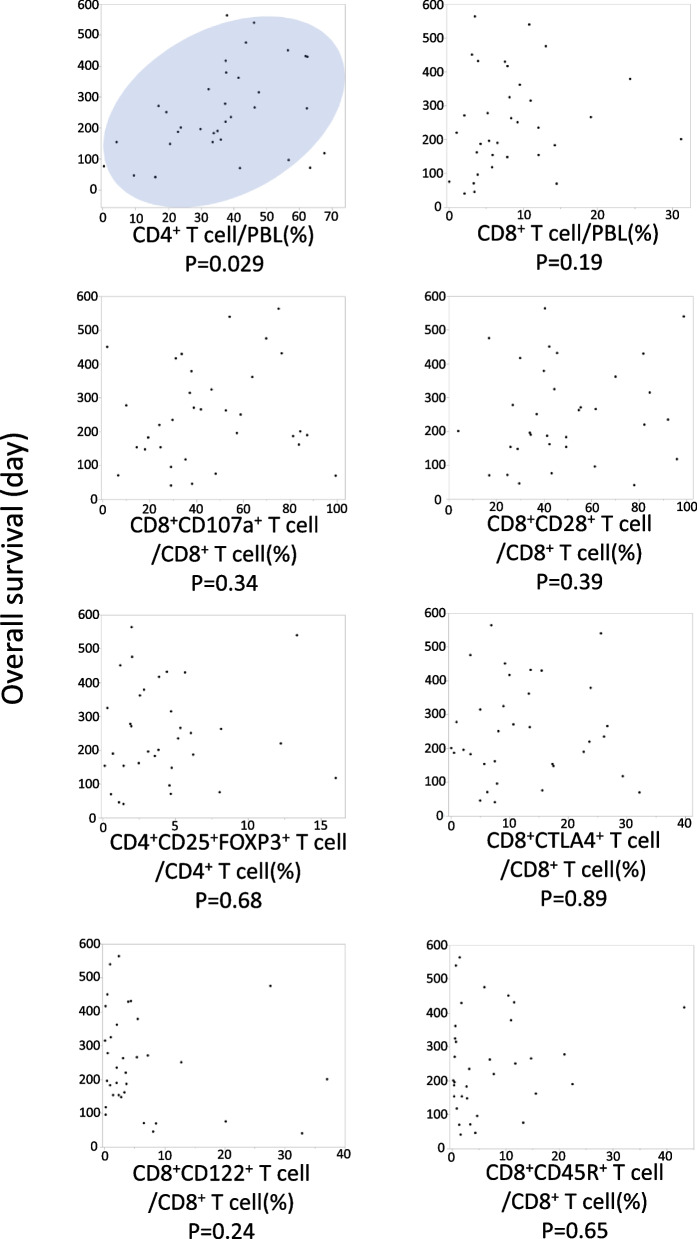
Correlation analysis between overall survival and across each subtype of peripheral blood lymphocyte (BPL) in patients with metastatic pancreatic cancer using Spearman’s rank correlation coefficient

Correlation analysis was performed to reveal the relationship between the PBLs of patients with MPC before chemotherapy (*n* = 34) and overall survival (OS). The proportion of CD4^+^ T cells in the PBLs of patients with MPC before chemotherapy positively correlated with OS (Fig. 2). Multivariate analysis was performed using the Cox multivariate proportional hazard regression model with the clinical prognostic factors (Table 2). Diabetes mellitus (*p* = 0.0076), serum albumin level (*p* = 0.030), Eastern Cooperative Oncology Group Performance Status (ECOG PS) (*p* = 0.0007), serum CA19–9 level (*p* = 0.0028), and peripheral CD4^+^ T cell ratio (*p* = 0.011) were significantly associated with prognosis in multivariate analyses.

**Table 2:** Univariate and multivariate analyses of prognostic factors in patients with metastatic pancreatic cancer

Covariate (***n***=54)	Univariate			Multivariate		
	Hazard ratio	95% CI	***P***-value	Hazard ratio	95% CI	***P***-value
Sex (Male)	1.51	0.75–3.07	0.25			
Age (≥65 years)	1.80	0.83–3.93	0.14			
Diabetes mellitus (yes)	0.70	0.34–1.42	0.32	0.34	0.15–0.75	**0.0076**
Serum albumin (g/dL)						
≥3.5	1.22	0.56–2.66	0.61	2.83	1.11–7.26	**0.030**
ECOG performance status						
1–2/0	2.02	0.99–4.13	0.054	4.67	1.92–11.32	**0.0007**
1st line chemotherapy						
mFFX/GnP	0.80	0.30–2.11	0.65			
GnP/S-IROX	0.81	0.11–6.14	0.84			
mFFX/S-IROX	0.65	0.08–5.74	0.70			
T factor						
4/1–3	0.52	0.24–1.14	0.10			
CA19-9 level (U/mL)						
≥1000	2.90	1.37–6.12	**0.005**	3.29	1.51–7.18	**0.0028**
CD4+ T cell level (%)						
High	0.45	0.22–0.92	**0.030**	0.35	0.15–0.78	**0.011**

*CI* confidence interval, *ECOG* Eastern Cooperative Oncology Group, *GnP* gemcitabine plus nab-paclitaxel, *SD* standard deviation, *mFFX* modified FOLFIRINOX

*P values <* 0.05 are shown in **bold**

**Fig. 3:**
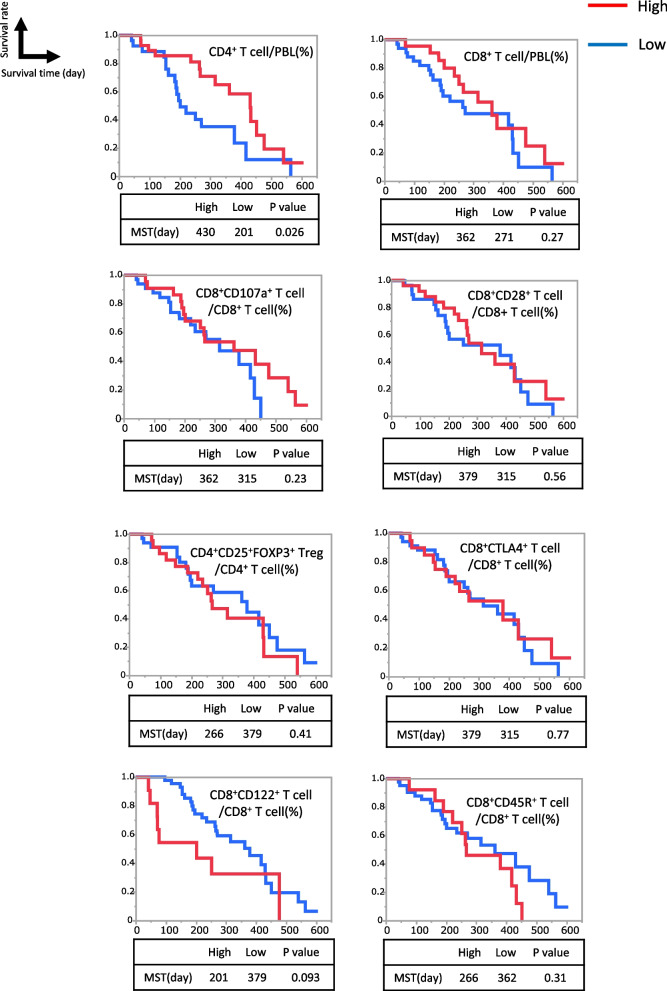
Kaplan–Meier analyses of survival between high and low groups for each subtype of peripheral blood lymphocytes using the log-rank test

Additionally, the cutoff value between the high and low groups for each PBL subtype was defined according to the mean value of the PBL ratio of MPC, as shown by the mean value of BPC in Online Resource 4. In the Kaplan–Meier method, the high level of CD4^+^ T cells before chemotherapy predicted significantly longer survival with the log-rank test. However, the other subtypes of PBLs before chemotherapy including the tumor-suppressive subtype (CD8^+^CD107a^+^ T cells, CD8^+^CD28^+^ T cells) and tumor-promoting subtypes (CD4^+^CD25^+^FOXP3^+^ Tregs, CD8^+^CTLA4^+^ T cells, CD8^+^CD122^+^ T cells, CD8^+^CD45R^+^ T cells) showed no significant correlation with the prognosis of patients with MPC both in correlation analysis and in the Kaplan–Meier method with log-rank test (Fig. 3).

### High proportion of CD8^+^CD122^+^ T cell was associated with short survival time of MPC

The low CD4^+^ T cell group showed a significantly shorter median survival time (MST) (201 days) than the high group (430 days, Fig. 3). Similarly, the high CD8^+^CD122^+^ T cell group had the same MST (201 days). Both Kaplan–Meier curves notably decreased at an early time course (< 200 days), and many events occurred < 90 days in the high CD8^+^CD122^+^ T cell population. This indicates the possible influence of peripheral CD4^+^ and CD8^+^CD122^+^ T cells on the early prognosis of patients with MPC. The patients were divided into two groups based on their survival time to determine the relevance of these two cell types.

**Fig. 4:**
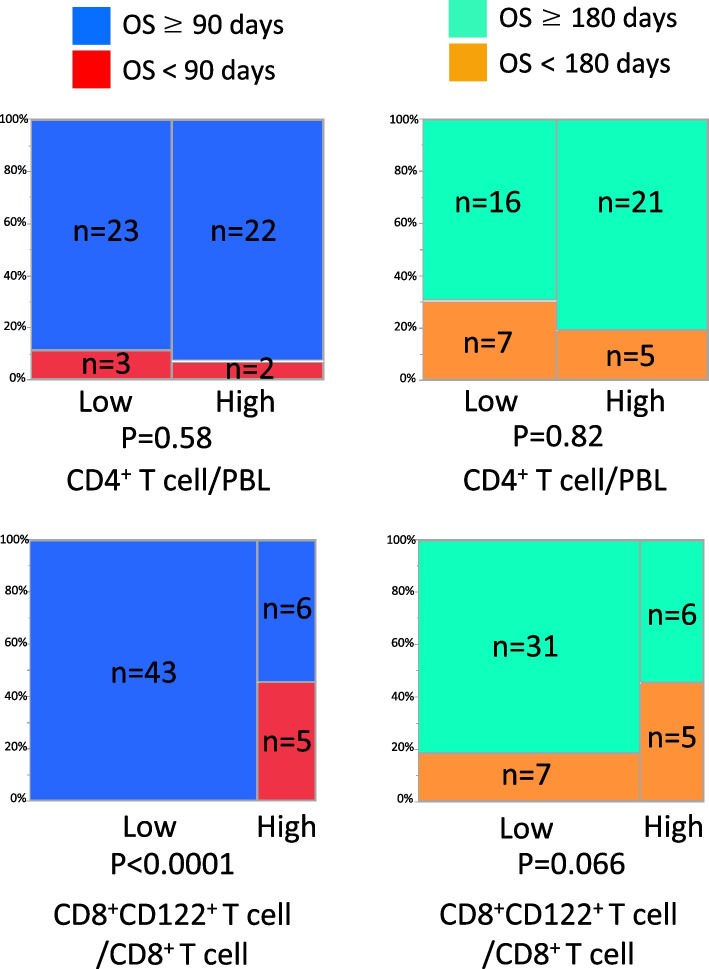
Correlation of the high and low group of peripheral CD4^+^ T cell or CD8^+^CD122^+^ T cell with short time survival(< 90 or < 180 days) using the chi-square test. Peripheral CD4^+^ T cells did not correlate with the early survival time (< 90 days, < 180 day) in patients with MPC (*P* = 0.58, *P* = 0.82). However, the high CD8^+^CD122^+^ T cell group was significantly associated with the short survival time with an obviously higher number of patients whose survival was < 90 days compared with the low CD8^+^CD122^+^ T cell group (*P* < 0.0001)

We setup the cutoff survival time at 90 and 180 days after chemotherapy induction. In Fig. 4, peripheral CD4^+^ T cells did not statistically correlate with the survival time in patients with MPC. In contrast, the high CD8^+^CD122^+^ T cell group was significantly associated with the short survival time, showing a remarkably higher number of patients whose survival was < 90 days compared with the low CD8 + CD122+ T cell group (0/43 vs 5/11, *p* < 0.0001). There were no significant differences in patient characteristics between the two groups of CD8^+^CD122^+^ T cells (Table 3). These results suggest that the proportion of CD8^+^CD122^+^ T cells before chemotherapy may be a prognostic indicator in patients with MPC.

**Table 3:** Clinical characteristics of patients with high and low CD8^+^CD122^+^ T cell levels

Characteristic	High CD8^**+**^CD122^**+**^ T cell ratio	Low CD8^**+**^CD122^**+**^ T cell ratio	***P***-value
Total number	11	43	
Sex (%)			
Male	4(36)	22 (51)	0.38
Female	7(64)	21(49)	
Age (years)			
Mean ± SD	62.7 ± 2.7	65.5 ± 1.4	0.50
Range	42–74	49–79	
Diabetes mellitus (%)			
Yes	2 (18%)	21 (49%)	0.67
Serum albumin (g/dL)			
Median	3.8	4.7	0.72
Range	2.2–4.3	2.6–4.5	
ECOG performance status (%)			
0/1/2	5(45.5)/5(45.5)/1(1)	26(61)/16(37)/1(2)	0.45
1^st^ line chemotherapy regimen (%)			
S-IROX/mFFX/GnP	1(9)/3(27)/7(64)	1(2)/8(19) /34(79)	0.43
T factor (%)			
1/2/3/4	0/0/5(45)/6(55)	1(2)/0/26(61)/16(37)	0.54
CA19-9 level (U/mL)			
Median	1924	571	0.21
Range	45.2–81446	1.5–606320	

*CI* confidence interval, *ECOG* Eastern Cooperative Oncology Group, *GnP* gemcitabine plus nab-paclitaxel, *SD* standard deviation, *mFFX* modified FOLFIRINOX

### CD8^+^CD122^+^ T cell ratio decreased in patients with MPC who benefited from chemotherapy

**Fig. 5:**
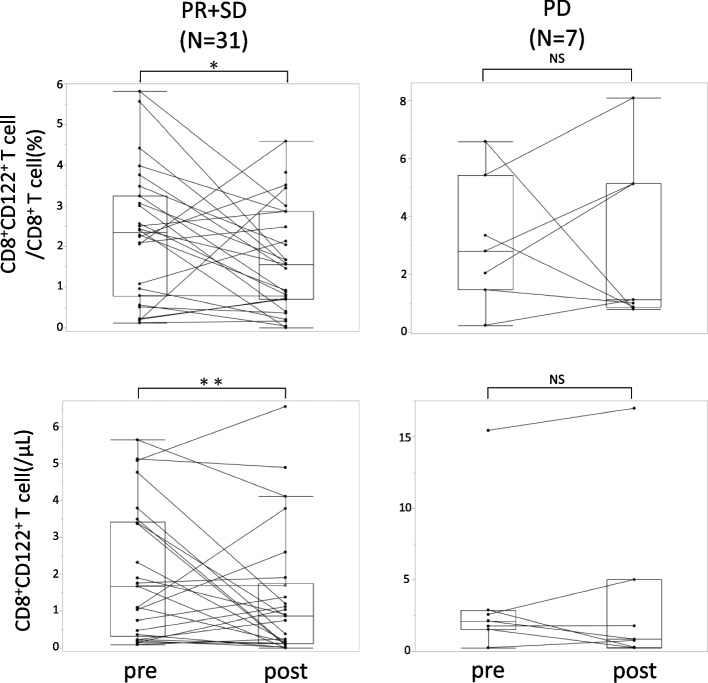
Comparison of the changes in CD8^+^CD122^+^ T cell after chemotherapy between PR + SD and PD groups using the paired t-test with the Wilcoxon signed-rank test. Pre: before chemotherapy, post: 2 months after chemotherapy induction. *P < 0.05, **P < 0.01, NS: not significant

The effect of chemotherapy can be classified as complete response (CR), partial response (PR), stable disease (SD), or progressive disease (PD) according to RECIST1.0. None of the patients with MPC showed CR in this study. Patients who show PR or SD could be regarded as receiving effective chemotherapy. We compared the distribution of CD8^+^CD122^+^ T cells in PR or SD cases with that in PD cases to investigate the relationship between CD8^+^CD122^+^ T cells and the efficacy of chemotherapy. The distribution of CD8^+^CD122^+^ T cells was evaluated before the introduction of chemotherapy and 2 months after. Consequently, CD8^+^CD122^+^ T cells significantly decreased both in proportion (*p* = 0.038) and number (*p* = 0.0032) in patients who showed PR or SD, while no obvious change was observed in patients with PD (Fig. 5). These results imply that chemotherapy can affect peripheral CD8^+^CD122^+^ T cells and alter their susceptibility to cancer. A comparison of the patient characteristics between the two groups is shown in Online Resource 5.

### Distribution of CD8^+^CD122^+^ T cell in TME depended on that in peripheral blood

**Fig. 6:**
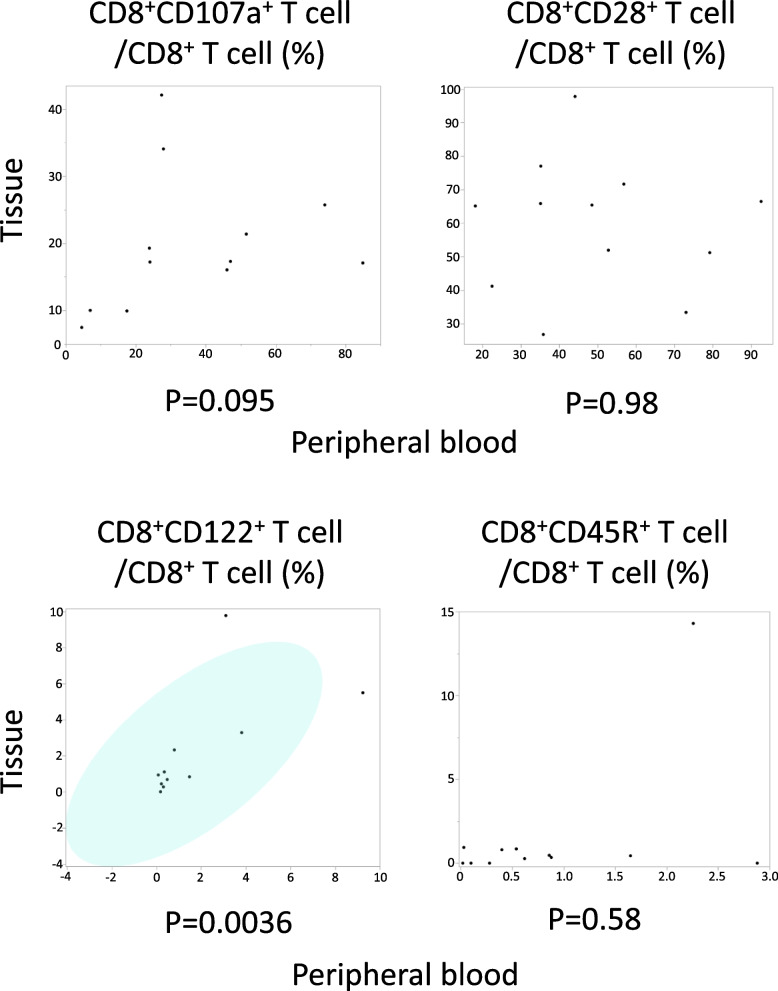
Correlation between the proportion of tissue infiltrating lymphocytes and those in peripheral blood using Spearman’s rank correlation coefficient

To determine the distribution of TILs in the TME and to clarify the correlation between TILs and PBLs, we collected blood and resected tissue samples from patients with RPC. Patient characteristics are shown in Online Resource 6. CD8^+^CD107a^+^ T cells, CD8^+^CD28^+^ T cells (tumor-suppressive subtypes), and CD8^+^CD122^+^ T cells (tumor-promoting subtype) were detected in both TME and peripheral blood (Fig. 6). However, CD8^+^CD45R^+^ T cells (tumor-promoting subtype) were observed in the peripheral blood but not in the TME. The proportion of CD8^+^CD122^+^ T cells in the TME was significantly correlated with that in peripheral blood (*p* = 0.0036), suggesting a direct link between the two. This result indicates that some TILs may circulate in the peripheral blood, and the distribution of PBLs may partially reflect the immunological microenvironment.

## Discussion

A comparison of the distribution of PBLs between patients with MPC and BPC showed elevated levels of both tumor-suppressive and tumor-promoting populations in MPC compared with BPC. This indicates that there is a crucial difference in the systemic immune environment between patients with BPC and MPC. Moreover, the competitiveness of increased tumor-promoting cells against increased tumor-suppressive cells may result in dysfunction of antitumor immunity and evasion of tumor cells from immune surveillance in the severe progression phase of MPC. However, Xu et al. (2014) showed a lower level of tumor-suppressive CD8^+^CD28^+^ T cells in PBLs of patients with pancreatic cancer than in those with benign cystic regions of the pancreas [13]. They enrolled patients with RPC at an earlier stage than in our study, which may explain our differing results.

Here, the proportion of peripheral CD4^+^ T cells before chemotherapy induction positively correlated with the OS of patients with MPC. This result is consistent with a previous report that showed a positive correlation between peripheral CD4^+^CD45RO^+^ T cell levels and the survival of patients with inoperable or MPC [14]. While we did not find a relationship between peripheral CD4^+^ Tregs and the prognosis of patients with MPC, previous studies have reported high levels of circulating CD4^+^ Tregs, leading to poor prognosis in patients with pancreatic cancer [13, 15]. This discrepancy might be the result of differences in the definitions of CD4^+^ Tregs; we defined them as CD4^+^CD25^+^FOXP3^+^ T cells, whereas CD4^+^ Tregs in the other reports are referred to as CD4^+^CD25^+^CD127^−^ T cells. In addition, we could not prove the predictability of tumor-suppressive subtypes of peripheral CD8^+^ T cells (CD107a^+^, CD28^+^), while other studies demonstrated that a high frequency of CD8^+^ or CD8^+^CD28^+^ T cells predicted better prognosis [13, 15]. This might be due to our smaller sample size, as each Kaplan–Meier curve of the high level of whole CD8^+^ or CD8^+^CD107a^+^ T cells showed a better survival rate than the low level, although the difference was not statistically significant.

Regulatory CD8^+^ T cells can be classified into several subtypes [29]. CD8^+^CD45R^+^ T cells have been classically regarded as a regulatory CD8^+^ T cell subtype because it was shown that they had a function of suppressing other effector cells in vitro [30, 31]. Memory CD8^+^CD122^+^ T cells have recently attracted considerable interest as a major regulatory CD8^+^ T cell subtype worldwide. Rifa’I et al. (2004) first reported that CD8^+^CD122^+^ T cells maintain T cell homeostasis by regulating other activated CD4^+^ and CD8^+^ T cells [32]. In some recent studies, CD8^+^CD122^+^ T cells showed immunosuppressive function, restrained autoimmune diseases [18, 19], and modulated allograft rejection or GVHD during organ transplantation [23, 25–27]. Other regulatory subtypes of CD8^+^ T cells, such as CD8^+^FOXP3^+^, CD8^+^CD28^−^ and CD8^+^CCR7^+^CD45RO^+^ have been reported [17, 29]. However, the roles and functions of CD8^+^CD45R^+^ T cells, CD8^+^CD122^+^ T cells, and others in the presence of cancer are mostly unknown. Thus, we selected CD8^+^CD45R^+^ T cells as the classical subtype and CD8^+^CD122^+^ T cells as the recent subtype to investigate their role in pancreatic cancer.

Our study demonstrated that patients with MPC had a higher proportion of both CD8^+^CD45R^+^ and CD8^+^CD122^+^ T cells in PBLs than in those with BPC. In addition, we found that the high level of CD8^+^CD122^+^ T cells in PBLs before chemotherapy was associated with early mortality (< 90 days) of patients with MPC, although no significant correlation was found between CD8^+^CD45R^+^ T cell subtype and prognosis. Previous studies have found that CD8^+^ T cells infiltrate the TME and inhibit the proliferation of other T cells [33, 34]. Moreover, anti-CD122 antibody reduces CD8^+^CD122^+^ T cells and suppresses tumor growth in colon cancer or melanoma in a murine model [33]. In a study using mice with hepatocellular carcinoma (HCC), resveratrol prevented HCC growth by blocking the differentiation of CD8^+^CD122^−^ T cells into CD8^+^CD122^+^ T cells [34]. In autoimmune diseases or immunosuppression during organ transplantation, CD8^+^CD122^+^ T cells suppressed other effector T cells by secreting IL-10 [18, 19, 23, 25, 26] or inducing apoptosis through Fas/FasL pathway [35, 36]. Therefore, in the presence of cancer, CD8^+^CD122^+^ T cells may prevent the activity of effector T cells, which impairs the progression of cancer, possibly by a mechanism similar to that previously reported.

Peripheral CD8^+^CD122^+^ T cells were reduced after chemotherapy induction in patients with PR or SD compared with those in patients with PD. Previous studies have revealed that peripheral CD4^+^FOXP3^+^ Tregs and MDSCs decreased after gemcitabine-based chemotherapy in patients with pancreatic cancer [37, 38]. Our study is the first to investigate the effect of chemotherapy on CD8^+^CD122^+^ T cells. We found that cancer chemotherapy may mediate cell fate decisions of CD8^+^CD122^+^ T cells and cause a subsequent change in susceptibility to chemotherapy. However, it was unclear whether the reduction in CD8^+^CD122^+^ T cells might result in increased susceptibility to chemotherapy or whether tumor shrinkage by chemotherapy might influence the fate of CD8^+^CD122^+^ T cells.

This is the first study to verify the distribution of CD8^+^CD122^+^ T cells both in the peripheral blood and the TME of patients with pancreatic cancer. As described above, the infiltration of CD8^+^CD122^+^ T cells into the TME depends on their proportion in the peripheral blood. CD8^+^CD107a^+^ and CD8^+^CD28^+^ T cells also exist in the TME, except for CD8^+^CD45R^+^ T cells. The presence of these T cells in the TME suggests that there is an interaction between these cells and cancer cells. Some T cells in the TME may migrate into the systemic blood circulation. Thus, we suggest that some CD8^+^CD122^+^ T cells in the TME may migrate into the peripheral blood circulation and peripheral CD8^+^CD122^+^ T cells level reflects immunosuppressive environment in the TME. Therefore, the high level of peripheral CD8^+^CD122^+^ T cells can predict poor prognosis in patients with MPC. However, in cancer research, the function of CD8^+^CD122^+^ T cells is still unknown. Therefore, further research should be desired.

Our findings suggest the possibility that CD8^+^CD122^+^ T cells may play a tumor-promoting role in pancreatic cancer. Therefore, they have potential to be new targets for antitumor therapy. However, most of their functions remain unknown and the sample size is small. Further investigation is required in the future.

## Supplementary Information

**Additional file 1.**

**Additional file 2.**

## Data Availability

The datasets supporting the conclusions of this article are included within the article and its additional files.
