# Supplementary material for: Circulating CD8+CD122+ T cells as a prognostic indicator of pancreatic cancer
Source: BMC Cancer. 2022 Nov 4;22:1134. doi: 10.1186/s12885-022-10207-0 (PMC9636831; doi:10.1186/s12885-022-10207-0)
Supplement: Supplementary file 1 — Additional file 1. [file 12885_2022_10207_MOESM1_ESM.pptx]

## Slide 1
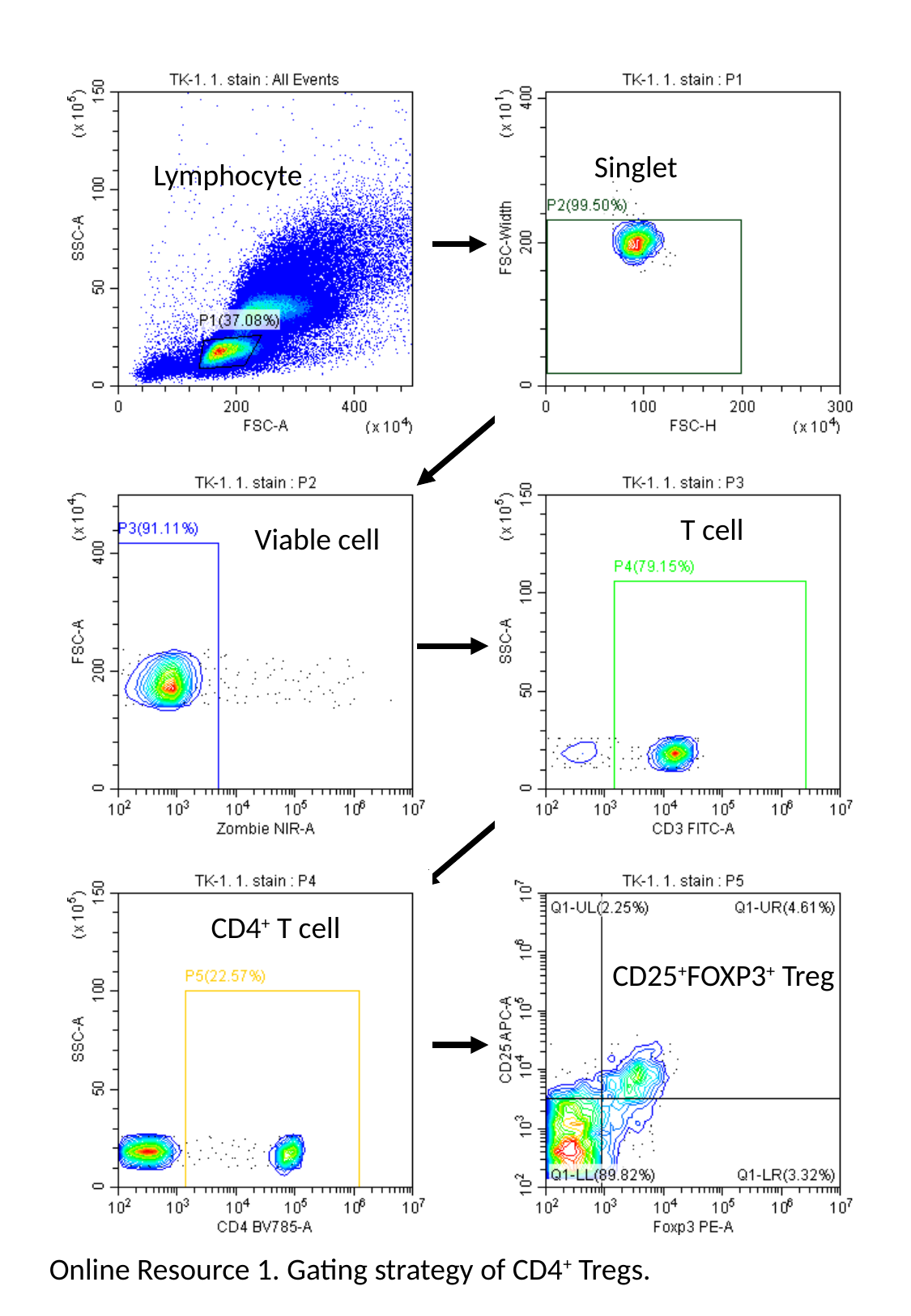

Lymphocyte
Singlet
Viable cell
T cell
CD4+ T cell
CD25+FOXP3+ Treg
Online Resource 1. Gating strategy of CD4+ Tregs.

## Slide 2
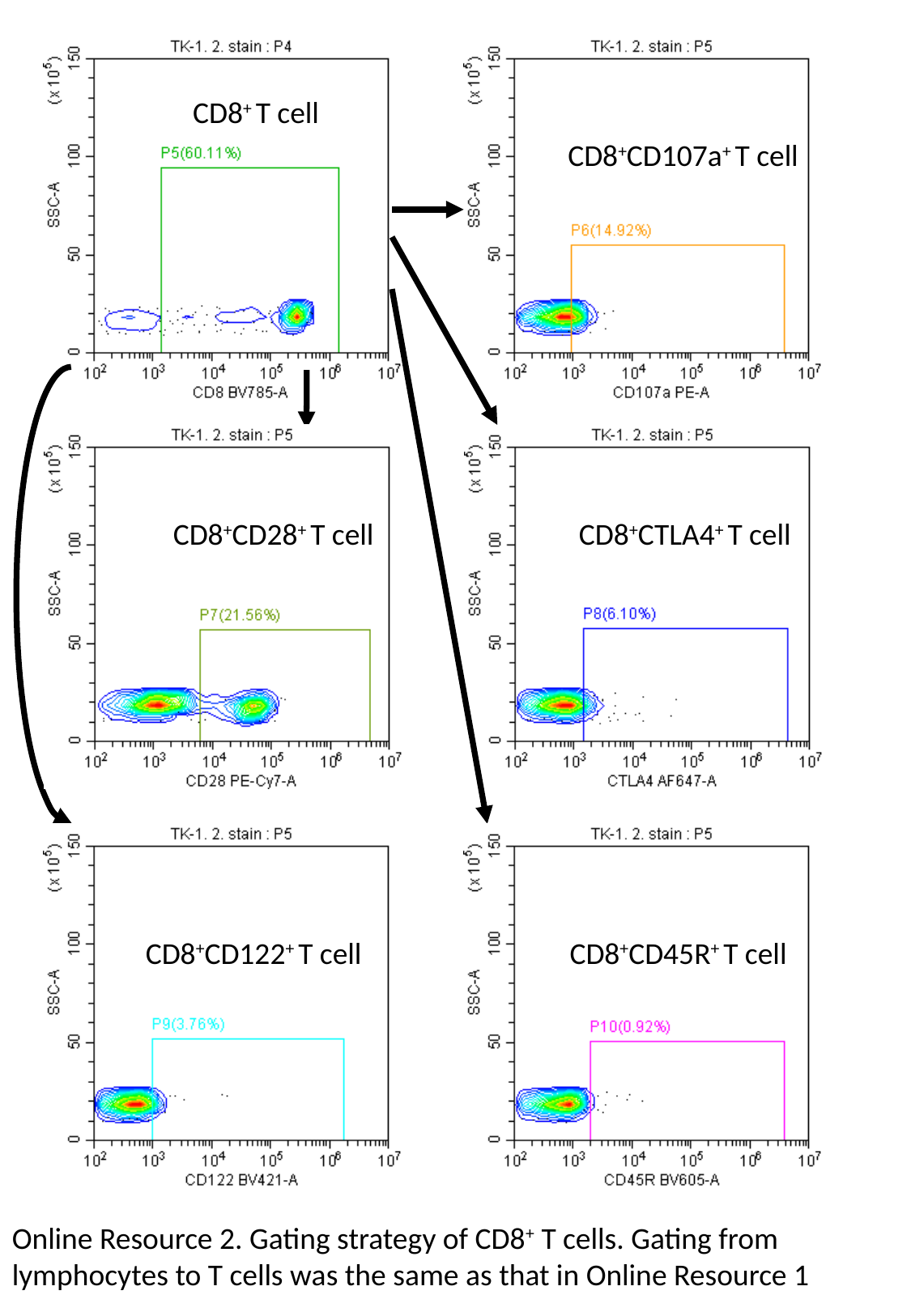

CD8+ T cell
CD8+CD107a+ T cell
CD8+CD28+ T cell
CD8+CTLA4+ T cell
CD8+CD122+ T cell
CD8+CD45R+ T cell
Online Resource 2. Gating strategy of CD8+ T cells. Gating from lymphocytes to T cells was the same as that in Online Resource 1

## Slide 3
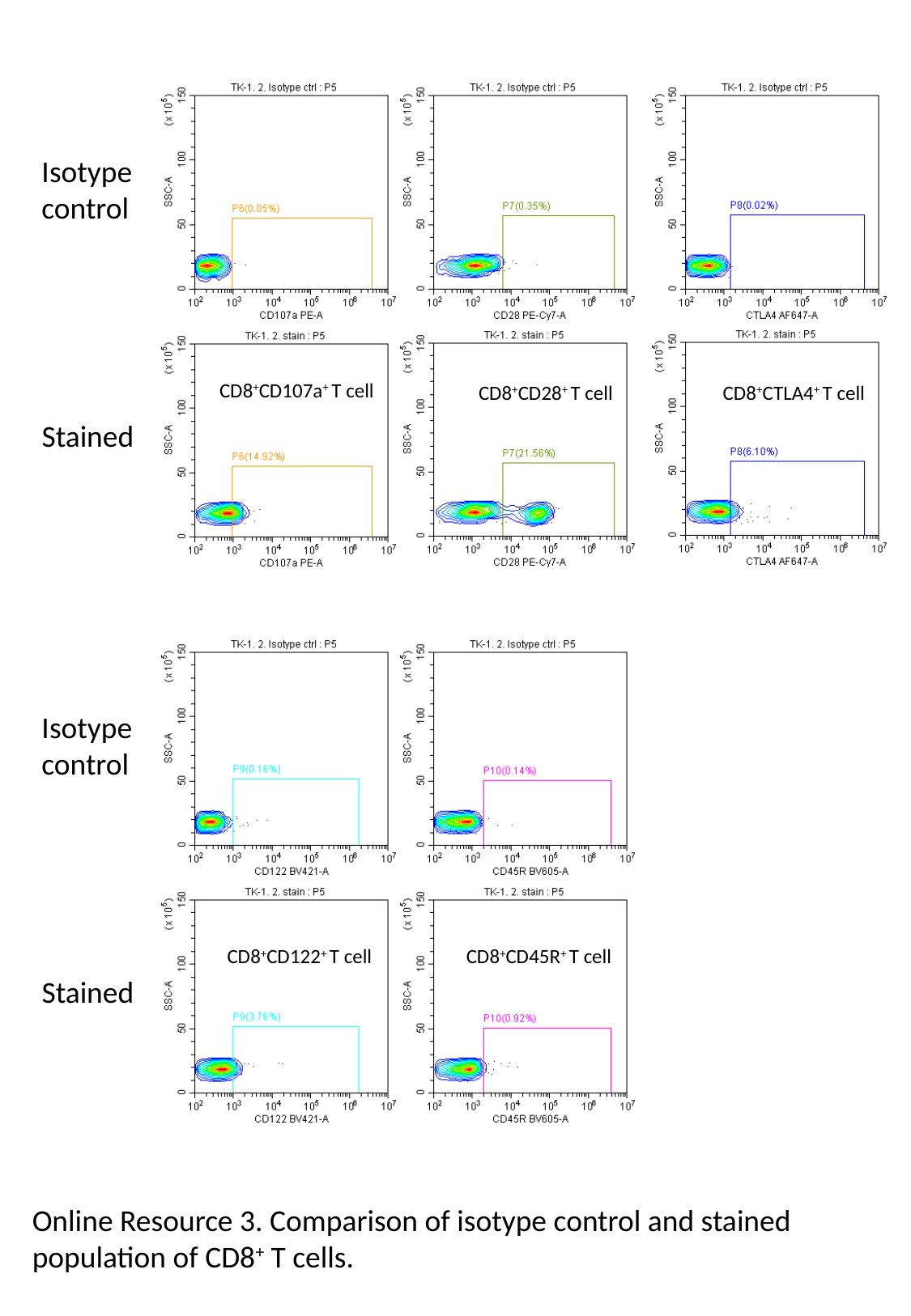

Isotype
control
CD8+CTLA4+ T cell
CD8+CD28+ T cell
CD8+CD107a+ T cell
Stained
Isotype
control
CD8+CD45R+ T cell
CD8+CD122+ T cell
Stained
Online Resource 3. Comparison of isotype control and stained population of CD8+ T cells.
