# Supplementary material for: Circulating CD8+CD122+ T cells as a prognostic indicator of pancreatic cancer
Source: BMC Cancer. 2022 Nov 4;22:1134. doi: 10.1186/s12885-022-10207-0 (PMC9636831; doi:10.1186/s12885-022-10207-0)
Supplement: Supplementary file 2 — Additional file 2. [file 12885_2022_10207_MOESM2_ESM.docx]

Online Resource 4. Mean proportions of each peripheral blood lymphocyte subtype in MPC and BPC The MPC value was adopted as the cutoff value between the high and low groups.

|  | CD4^+^ T cell | CD8^+^ T cell | CD8^+^CD107a^+^ T cell | CD8^+^CD28^+^ T cell | CD4^+^CD25^+^FOXP3^+^ Treg | CD8^+^CTLA4^+^ T cell | CD8^+^CD122^+^ T cell | CD8^+^CD45R^+^ T cell |
| --- | --- | --- | --- | --- | --- | --- | --- | --- |
| **MPC** | 39.07 | 8.69 | 39.56 | 49.17 | 4.16 | 13.84 | 6.14 | 4.79 |
| **BPC** | 31.22 | 8.77 | 17.24 | 30.8 | 2.79 | 8.15 | 3.23 | 0.59 |

MPC: metastatic pancreatic cancer, BPC: benign pancreatic cyst.

Online Resource 5. Clinical characteristics of patients with effective chemotherapy with partial progress (PR)/ stable disease (SDi), and progressive disease (PD)

| **Characteristic** | **PR/SDi** | **PD** | ***P*-value** |
| --- | --- | --- | --- |
| Total number | 31 | 57 |  |
| Sex (%) |  |  |  |
| Male | 16 (52) | 5 (71) | 0.87 |
| Female | 28 (52) | 27 (52) |  |
| Age (years) |  |  |  |
| Mean ± SD | 64.8 ± 1.4 | 62.6 ± 4.6 | 0.85 |
| Range | 42–74 | 49–79 |  |
| Diabetes mellitus (%) |  |  |  |
| Yes | 19 (47.5) | 2 (22.2) | 0.17 |
| Serum albumin (g/dL) |  |  |  |
| Median | 3.7 | 3.6 | 0.37 |
| Range | 2.6–4.5 | 3.5–4.5 |  |
| ECOG performance status (%) |  |  |  |
| 0/1/2 | 21(68)/9(29)/1(3) | 2(29)/5(71)/0 | 0.11 |
| 1^st^ line chemotherapy regimen (%) |  |  |  |
| S-IROX/mFFX/GnP | 2(7)/5(16)/24(77) | 0/3(43)/4(57) | 0.26 |
| T factor (%) |  |  |  |
| 1/2/3/4 | 1(3)/0/19(61)/11(36) | 0/0/6(86)/1(14) | 0.46 |
| CA19-9 level (U/mL) |  |  |  |
| Median | 494 | 200.7 | 0.76 |
| Range | 2.5–606320 | 1.5–103028 |  |

GnP: gemcitabine plus nab-paclitaxel, mFFX: modified FOLFIRINOX, PD: progressive disease, PR: partial response, SD: standard deviation, SDi: stable disease.

Online Resource 6. Clinical characteristics of patients with resectable pancreatic cancer

| **Characteristic** | **Value (n=12)** |
| --- | --- |
| Sex (%) |  |
| Male | 7 (58.3) |
| Female | 5 (41.7) |
| Age (years) |  |
| Mean ± SD | 67.6±12.5 |
| Range | 40–82 |
| Diabetes mellitus (%) |  |
| Yes | 8 (66.7%) |
| Serum albumin (g/dL) |  |
| Median | 4.1 |
| Range | 2.4–4.5 |
| Neoadjuvant chemotherapy regimen (%) |  |
| non/GEM+S-1/mFFX/GnP | 1(8.3)/ 3(25)/ 3(25)/ 5(41.7) |
| Location of tumor in pancreas (%) |  |
| Head/body/tail | 8(66.7)/ 2(16.65)/ 2(16.65) |
| T factor (%) |  |
| 1/2/3/4 | 0/ 0/ 12(100)/ 0 |
| Tumor size(mm) |  |
| Mean±SD | 26.8 ± 22.9 |
| Range | 9–90 |
| CA19-9 level (U/mL) |  |
| Median | 64.05 |
| Range | 2–6877 |

GnP: gemcitabine plus nab-paclitaxel, mFFX: modified FOLFIRINOX, SD: standard deviation.
